# Seawater pearl hydrolysate alleviates perimenopausal syndrome by modulating hypothalamic and uterine ERα/MAPK/CREB signaling in ovariectomized rats

**DOI:** 10.3389/fphar.2026.1749728

**Published:** 2026-02-05

**Authors:** Yasheng Deng, Tianwei Liang, Hui Huang, Siyin Han, Yanping Fan, Jiang Lin

**Affiliations:** 1 FangChengGang Hospital of Traditional Chinese Medicine, Fangchenggang, China; 2 Guangxi University of Chinese Medicine, Nanning, China; 3 Dongguan Hospital of Traditional Chinese Medicine, Dongguan, China

**Keywords:** CREB, ERα, estrogen receptor signaling, ethnopharmacology, MAPK, neuroendocrine regulation, ovariectomized rat model, perimenopausal syndrome

## Abstract

**Background:**

Perimenopausal syndrome (PMS), characterised by hormonal imbalance resulting from ovarian aging, causes various symptoms that significantly impair quality of life. Current hormone replacement therapy carries potential risks; thus, safer alternatives are needed. This study investigated the therapeutic efficacy and underlying mechanisms of seawater pearl hydrolysate (SPH) against PMS in an ovariectomized (OVX) rat model, focusing on the estrogen receptorα (Erα)/mitogen-activated protein kinase (MAPK)/cAMP-responsive element-binding protein (CREB) signaling pathway.

**Methods:**

A PMS rat model was established via bilateral ovariectomy. Rats were divided into Sham, Model, Kuntai capsule (KT, positive control), and low-, medium-, and high-dose SPH groups. After 15 days of treatment, we assessed estrous cycles, open-field behavior, serum sex hormones [estradiol (E_2_), progesterone (P), follicle-stimulating hormone (FSH), luteinizing hormone (LH), gonadotropin-releasing hormone (GnRH), testosterone (T), anti-Müllerian hormone (AMH), oxidative stress markers (malondialdehyde (MDA), superoxide dismutase (SOD)], lipid profiles [total cholesterol (TC), triglyceride (TG), low-density lipoprotein cholesterol (LDL-C), high-density lipoprotein cholesterol (HDL-C)], uterine histopathology, and mRNA/protein expression of key components [ERα, mitogen-activated protein kinase kinase 1 (MAP2K1), extracellular signal-regulated kinase (ERK), CREB] in uterine and hypothalamic tissues.

**Results:**

Compared with the sham group, model rats showed disrupted estrous cycles, decreased locomotor activity, reduced uterine and hypothalamic indices, abnormal tissue morphology, significantly reduced serum levels of E_2_ and P, and elevated levels of FSH, LH, and GnRH. SPH treatment, particularly at medium and high doses, reversed OVX-induced impairments in a dose-dependent manner. Specifically, SPH ameliorated behavioural deficits, restored estrous cycles, improved uterine histoarchitecture, and normalised serum hormone levels (E_2_, P, FSH, LH). Additionally, SPH reduced oxidative stress (MDA) and improved dyslipidaemia. Mechanistically, these therapeutic effects were associated with increased mRNA and protein expression (including phosphorylation) of ERα, MAP2K1, ERK, and CREB in uterine and hypothalamic tissues.

**Conclusion:**

SPH effectively alleviates PMS symptoms in OVX rats. Its therapeutic effects are associated with restored hormonal balance, reduced oxidative stress, improved lipid metabolism, and modulation of the ERα/MAPK/CREB signaling pathway in both uterine and hypothalamic tissues, suggesting an integrated neuroendocrine regulatory mechanism. Further studies employing functional inhibition experiments are warranted to confirm these causal relationships.

## 1 Introduction

Perimenopausal syndrome (PMS) refers to physiological and somatic changes in multiple tissues and organs caused by declining estrogen levels during the transition to menopause (Fu et al., 2024; Wang et al., 2025). Clinically, PMS is characterised by symptoms such as hot flashes, night sweats, menstrual irregularities, irritability, and depression. If not promptly and effectively managed, PMS can significantly increase the risk of postmenopausal osteoporosis, coronary heart disease, and diabetes (Erdélyi et al., 2023). Epidemiological studies indicate that approximately one billion women worldwide experience PMS, with 47 million new cases reported annually, highlighting it as a major public health issue (Sourouni et al., 2021; Santoro, 2016). Current clinical strategies aim primarily to relieve symptoms and improve quality of life rather than delay physiological aging (Nappi et al., 2023). Although hormone replacement therapy (HRT) is effective in alleviating symptoms, its long-term use increases risks of breast cancer, endometrial cancer, and thromboembolic events. Therefore, the development of precise, effective, and low-risk non-hormonal therapies has become an important research priority to support the health of menopausal women.

In response to this therapeutic challenge, researchers have investigated safe and effective non-hormonal alternatives. Pearl has a long history of medicinal use, initially recorded in Lei Gong’s Treatise on Drug Processing and elaborated upon in the Compendium of Materia Medica. It is traditionally used to “calm the ethereal and corporeal souls”, addressing common PMS symptoms such as anxiety and insomnia. Additionally, it is believed to “stop seminal emission”, reflecting its role in consolidating kidney essence. This aligns with the traditional Chinese medicine (TCM) understanding of PMS, which is associated with kidney yin deficiency. Modern clinical observations have confirmed that pearl-based treatments effectively manage PMS without reported adverse effects (Zhang et al., 2019; Fang et al., 2022).

Our research group has developed a novel SPH preparation through a proprietary enzymatic hydrolysis method. This innovative formulation represents a significant advancement in ethnopharmacology. The process is designed to preserve and potentially enhance the bioavailability of bioactive components from pearl and honey. Preliminary studies demonstrated that this formulation has pharmacological effects, including promoting vascular endothelial cell proliferation (Cen et al., 2018) and reducing oxidative stress damage (Liu et al., 2020). These findings provide a theoretical basis for exploring its therapeutic mechanisms in PMS treatment. Mitogen-activated protein kinase kinase 1 (MAP2K1) is a critical kinase in the extracellular signal-regulated kinase (ERK) signaling pathway. This pathway regulates cell proliferation and differentiation by mediating the nuclear translocation of ERK proteins (Sun et al., 2015). Declining estrogen levels reportedly suppress the MAPK/ERK signaling pathway, causing abnormal expression of downstream target proteins. Consequently, this creates a vicious cycle that exacerbates endocrine dysfunction (Kawvised et al., 2017).

**FIGURE 1:**
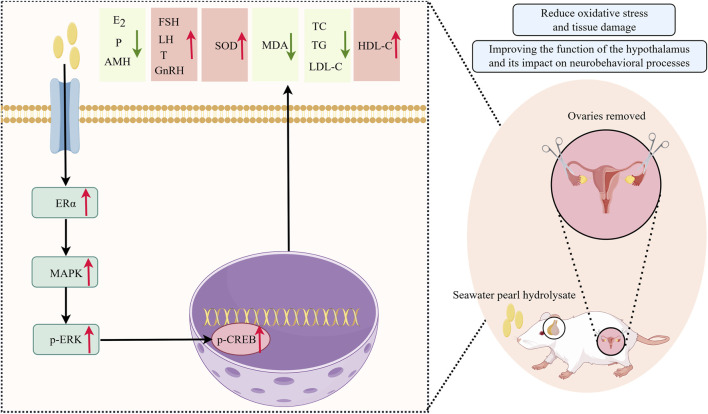
Graphical abstract (drawn by Figdraw).

To bridge the gap between traditional use and modern mechanistic understanding, contemporary methods such as network pharmacology have proven valuable in deciphering the multi-target actions of natural products. Examples include studies of naringenin (Zhou et al., 2024) and Tanshinone IIA (Zhou et al., 2025). Advanced molecular network analyses have elucidated critical pathways in reproductive endocrine disorders (Zheng et al., 2025; Wu et al., 2023). Detailed investigations of estrogen receptor signaling, particularly ESR2-linked axes, have refined understanding of neuroendocrine regulation in affective and reproductive health (Luo et al., 2024). Inspired by these insights, this study systematically evaluates the effects of SPH on serum estrogen levels, uterine histomorphology, and key proteins (MAPK/CREB) signaling pathways using an ovariectomized (OVX) rat model. The goal is to elucidate the specific molecular mechanisms underlying SPH’s therapeutic effects on PMS and to provide scientific evidence supporting the development of novel therapeutic agents for PMS. The overall experimental design is summarized in Figure 1.

## 2 Materials and methods

### 2.1 Source, authentication, and ethical sourcing of seawater pearl material

The seawater pearls used in this study were sourced from cultured *Pinctada martensii* mussels, a common species used in freshwater pearl aquaculture in China. The pearls were not collected from wild populations but were commercially obtained as a by-product of sustainable aquaculture practices. The material was purchased from Beihai Baozhulin Marine Technology Co., Ltd. (Guangxi, China), a licensed supplier. Species identification and material authentication were performed by Dr. Jiang Lin (co-author, Guangxi University of Chinese Medicine), a specialist in marine medicinal materials, based on morphological characteristics (nacreous layer structure, luster, shape typical of cultured pearls from this species) and supplier documentation. A voucher specimen of the raw pearl powder (batch number: 017BHR017) has been deposited at the Herbarium of Marine Medicinal Materials, Guangxi University of Chinese Medicine, Nanning, China (Accession No. GXUCM-017BHR017). The procurement and use of this cultured material comply with relevant national regulations in China and do not involve species listed under CITES, the IUCN Red List as threatened, or other protection categories. The sourcing is considered ethical and sustainable, as it utilizes a product from established aquaculture.

### 2.2 Animals

Sixty specific pathogen-free (SPF) female Sprague-Dawley (SD) rats (2 months old, weighing 220 ± 20 g) were purchased from Hunan Silaike Jingda Laboratory Animal Co., Ltd. (Production License: SCXK [Xiang] 2019–0004). The animals were housed in the Animal Research Center of Guangxi University of Chinese Medicine (Facility License: SYXK [Gui] 2019–0001) under controlled conditions: temperature 23 °C ± 3 °C, relative humidity 55% ± 5%, and a 12-h light/dark cycle. Rats were provided *ad libitum* access to standard rodent chow and water. The experiment was initiated after a 1-week acclimatization period. All experimental procedures were approved by the Institutional Animal Care and Use Committee of Guangxi University of Chinese Medicine (Ethics Approval No.: DW20230411-074).

### 2.3 Drugs and main reagents

The drugs and primary reagents used in this study, including their manufacturers, sources, and batch/item numbers, are listed in Table 1.

**TABLE 1:** Drugs and main reagents.

Reagent name	Manufacturer/Item number	Lot/Catalog number
Seawater pearl powder (cultured *Pinctada martensii*)	Beihai baozhulin MarineTechnology Co., ltd	450522020014
Kuntai capsules	Guiyang xintian pharmaceutical Co., ltd	220903
Penicillin sodium for injection	Shandong shengwang pharmaceutical Co., ltd	20211101
Neutral protease	Beijing Solarbio science & technology Co., ltd	3424080827
Follicle-stimulating hormone (FSH), luteinizing hormone (LH), estrogen (E_2_), testosterone (T), progesterone (P) and (anti-müllerian hormone) AMH ELISA kits	Shanghai Jianglai biotechnology Co., ltd.; jiangsu enzyme immunoassay industrial Co., ltd	JL13251、JL21049、JL11473、MM-0219R6、MM-0219R1、MM-0575R11
Total cholesterol (TC), triglycerides (TG), low-density lipoprotein cholesterol (LDL-C), high-density lipoprotein cholesterol (HDL-C) ELISA kits	Nanjing jiancheng technology Co., ltd	A111-1-1、A110-1-1、A113-1-1、A112-1-1
Tumor necrosis factor-α (TNF-α), Interleukin-1β (IL-1β), Interleukin-6 (IL-6) ELISA kits	Nanjing jiancheng technology Co., ltd	H052-1-1、H002-1-2、H007-1-1
Malondialdehyde (MDA), superoxide dismutase (SOD), glutathione peroxidase	W nanjing jiancheng technology Co., ltd	A003-1-2、A001-3-2
TRIpure total RNA extraction reagent	ELK biotechnology Co., ltd	EP013
Hematoxylin and eosin (H&E) staining kit	Beijing Solarbio science & technology Co., ltd	C0105
Reverse transcription kit, quantitative real-time PCR kit	Wuhan aspen biotechnology Co., ltd	MR05101、MQ00701
RIPA lysis buffer, BCA protein assay kit, phosphatase inhibitor cocktail, ECL chemiluminescence detection kit; HRP-conjugated goat anti-rabbit/anti-mouse secondary antibodies	Wuhan aspen biotechnology Co., ltd	AS1004、AS1086、AS1008、AS1059、AS1114、AS1093
ERα, GAPDH primary antibodies	Abcam (shanghai) trading Co., ltd	ab32063、ab181602
MAP2K1 primary antibody	Wuhan sanying biotechnology Co., ltd	67872-1-Ig
p-ERK, p-CREB primary antibodies	Cell signaling technology (CST)/Shanghai nuoning biotechnology Co., ltd	4370、9198

### 2.4 Instruments and equipment

The main instruments and equipment employed in this study are summarised in Table 2.

**TABLE 2:** Instruments and equipment.

Instrument name	Manufacturer/Model	Model
Multifunctional microplate reader	Xihuavedelang instruments Co., ltd	DR-200Bs
High-speed refrigerated centrifuge	Hunan xiangyi laboratory instrument development Co., ltd	TGL-16
Tissue dehydrator, embedding machine	Wuhan junjie electronics Co., ltd	JT-12J、JB-P5
Pathology microtome	Shanghai Leica instruments Co., ltd	RM2016
Optical microscope	Olympus corporation (shanghai)	CX21
Real-time quantitative PCR system	Applied biosystems (ABI), USA	ABI7500
Clean bench	Jinan XINBIXI biotechnology Co., ltd	BSC-1100IIA2-X
Electrophoresis apparatus, transfer electrophoresis tank, vertical electrophoresis tank	Beijing Liuyi instrument Co., ltd	DYY-6C、DYCZ-400D、DYCZ-24DN

### 2.5 Preparation of SPH

SPH, the active ingredient used in this study, was prepared from authenticated seawater pearl powder (from cultured *Pinctada martensii*) through a standardised enzymatic hydrolysis process designed to solubilize organic matrix components and release bioactive peptides and trace elements, moving beyond a simple calcium carbonate powder. Briefly, raw pearls were cleaned, dried, and pulverised into fine powder using a ball mill. Powder particles of uniform size (≤45 μm) were obtained through sieving. The pearl powder was suspended in deionised water at a solid-to-liquid ratio of 1:20 (w/v). The suspension pH was adjusted to 7.5, and neutral protease (1.5 × 10^4^ U/g) was added (1000 U per Gram of substrate) to initiate hydrolysis. The reaction mixture was incubated at 50 °C for 8 h in a shaking water bath. Hydrolysis was terminated by heating at 90 °C for 10 min to inactivate the enzyme. The hydrolysate was centrifuged (8,000 × g, 15 min, 4 °C) to remove insoluble residues. The clear supernatant containing soluble peptides and trace elements was collected and lyophilised to obtain the final SPH product. The degree of hydrolysis and peptide yield were regularly monitored to ensure batch-to-batch consistency.

### 2.6 Analysis of proteins and trace elements in SPH

Protein analysis was performed by liquid chromatography-mass spectrometry (LC-MS). Briefly, proteins were extracted from the SPH sample using RIPA Lysis and Extraction Buffer. Extracted proteins were separated using SDS-PAGE. Following electrophoresis, the gel lanes were excised and destained. The destained gel fragments were digested in-gel with trypsin. The resulting peptide mixtures were analysed by LC-MS to obtain raw spectral data. Data processing and protein identification were performed using MaxQuant software (version 1.6.2.10, Max Planck Institute of Biochemistry).

For trace element analysis, approximately 0.5 g of SPH was weighed and placed into a microwave digestion vessel. After adding 5 mL nitric acid, the sealed vessel underwent microwave-assisted digestion. After cooling to room temperature, the digestate was diluted to a final volume of 25 mL using ultrapure water. The concentrations of iron (Fe), manganese (Mn), copper (Cu), sodium (Na), magnesium (Mg), zinc (Zn), selenium (Se), arsenic (As), and mercury (Hg) were determined by atomic fluorescence spectrometry. Levels of sulfur (S), phosphorus (P), and strontium (Sr) were measured by inductively coupled plasma atomic emission spectrometry (ICP-AES).

### 2.7 Grouping, modeling, and dosing of rats

The PMS rat model was established by bilateral OVX according to established protocols (Park et al., 2022). Surgical procedures were performed as follows: after 12 h of fasting, rats were weighed and anesthetized via intraperitoneal injection of 1% sodium pentobarbital (30 mg/kg). Animals were placed in a ventral recumbency position. The surgical site was shaved approximately 2 cm lateral to the spine along the mid-axillary line below the last rib and disinfected with iodine. An incision (1.5–2.0 cm) was made through the skin and dorsal muscles to access the peritoneal cavity. The ovaries embedded in white adipose tissue were carefully exposed using forceps. After separating surrounding fat, the pink follicular ovaries were identified. The fallopian tubes were ligated, and both ovaries were completely excised. The wound was closed in layers and disinfected with iodophor. In the Sham-operated group, identical procedures were performed except that only part of the periovarian fat was removed, leaving the ovaries intact. Postoperative care included intramuscular injection of penicillin sodium (50,000 U/kg/day) for three consecutive days to prevent infection. Vaginal smears were collected daily for 5 days, starting on postoperative day 6, following a brief adaptation period, to monitor estrous cycles. Successful model establishment was confirmed by persistent diestrus, indicating the absence of estrus-phase cells.

The doses of SPH (0.0315, 0.063, and 0.126 g/kg/day) were selected based on a combination of traditional usage equivalents. The middle dose (0.063 g/kg/day) was converted from the typical human clinical dosage (adjusted by body surface area), while the low and high doses were set at 0.5- and 2-fold of the middle dose, respectively, to evaluate a potential dose-response relationship. This range was further supported by our pilot study, which indicated significant biological activity without observable toxicity within these doses.

Forty-eight qualified rats were randomly allocated to six groups (n = 8 per group): Sham group (sham operation + distilled water), Model group (OVX + distilled water), KT group (OVX + Kuntai capsules, 0.63 g/kg/day), and three SPH groups, SPHL, SPHM, and SPHH (OVX + SPH at 0.0315, 0.063, and 0.126 g/kg/day, respectively). These group abbreviations were used consistently in subsequent analyses. Drug treatments began on postoperative day 10 and continued for 15 days, administered daily by oral gavage (10 mL/kg/day).

### 2.8 General observation of rats

Body weight was recorded daily at fixed times. After the final administration, rats were fasted for 12 h with free access to water and anesthetized via intraperitoneal injection of 2% sodium pentobarbital. Blood samples were collected from the abdominal aorta, and serum was isolated by centrifugation (3,000 rpm, 15 min) and stored at −80 °C. The ovaries and uterus were carefully dissected, and surrounding adipose tissues were removed. After weighing, parts of uterine tissues were fixed in 4% paraformaldehyde for histomorphological examination, while remaining tissues were snap-frozen and stored at −80 °C for PCR and Western blot analyses.

### 2.9 Open field test (OFT)

One hour prior to the open field test, rats were acclimated to the testing chamber environment. Each rat was gently placed at the center of a 100 cm × 100 cm open field arena, with its head oriented consistently. Animal behavior analysis software automatically recorded rat activity within the arena for 5 min. Following each session, urine and feces were promptly removed, and the arena was thoroughly cleaned with 75% ethanol solution to eliminate odor residues and prevent interference. Parameters recorded included average movement speed, total distance traveled, and activity time during the 5-min observation period.

### 2.10 Determination of uterine and hypothalamic indices

Wet weights of the uterus and hypothalamus were measured for all experimental groups. Organ indices were calculated using the following formulas: Uterine index (%) = [uterine wet weight (mg)/body weight (g)] × 100%; Hypothalamic index (%) = [hypothalamic wet weight (mg)/body weight (g)] × 100%

### 2.11 HE staining

Vaginal smears and uterine tissues were examined by hematoxylin-eosin (HE) staining. For vaginal cytology, air-dried smears were fixed in 95% ethanol for 5 min, stained with hematoxylin for 7–10 min, and rinsed 3–5 times. Smears were differentiated in 1% hydrochloric acid for 5–10 s, rinsed for 1 min, counterstained with eosin for 4–6 min, and rinsed again 2–3 times. Samples were cleared in xylene for 5–7 min, air-dried, and mounted with neutral balsam. Uterine tissues were fixed for 24 h, processed through graded ethanol dehydration, xylene clearing, and paraffin embedding. Sections (4–5 μm) were dewaxed, hydrated, and stained according to standard HE staining protocols, then dehydrated, cleared, and mounted for morphological evaluation under light microscopy.

### 2.12 ELISA

Serum samples were centrifuged at 3,000 × g for 10 min (radius: 10 cm) to obtain supernatants. ELISA assays were performed following manufacturers’ protocols to measure serum reproductive hormones (E_2_, FSH, LH, GnRH, T, P, AMH) and oxidative stress markers (MDA, SOD). After terminating reactions, optical density (OD) values were measured using a microplate reader. Standard curves were generated using provided calibrators, and analyte concentrations were calculated by curve-fitting analysis.

### 2.13 RT-qPCR

Total RNA was extracted from uterine and hypothalamic tissues using TRIpure reagent and reverse-transcribed into cDNA. Quantitative real-time PCR (RT-qPCR) was performed using the SYBR Green detection system to quantify mRNA expression levels of ERα, MAPK, ERK1/2, and CREB, with β-actin as an internal reference gene. Primer sequences for target genes were synthesized by Wuhan Hengyi Sai Biotechnology Co., Ltd. (sequences provided in Table 3). Relative gene expression levels were determined using the ΔCt method and calculated using the 2^−ΔΔCt^ formula.

**TABLE 3:** Specific information on primer sequence design.

Primer	Sequence
ERα	sense:5′-CAGTGAAGCCTCAATGATGGG-3′ antisense:5′-AGAAGGTGAACTTGATCGTGGAG-3′
Map2k1	sense:5′-GATCAAGTGCTGAAGAAAGCTG-3′ antisense:5′-CTAGAATGTTGGAAGGCTTGACAT-3′
ERK1	sense:5′-CTGGCTTTCTGACCGAGTATGT-3′ antisense:5′-AATTTAGGTCCTCTTGGGATGG-3′
ERK2	sense:5′-GCACCAACCATTGAGCAGAT-3′ antisense:5′-TCACGGTGCAGAACATTAGCT-3′
CREB	sense:5′-CCCAGGGAGGAGCAATACAG-3′ antisense:5′-GGTGCTGTGCGAATCTGGTAT-3′
β-actin	sense:5′-CGTTGACATCCGTAAAGACCTC-3′ antisense:5′-TAGGAGCCAGGGCAGTAATCT-3′

### 2.14 Western blot

Appropriate amounts of uterine and hypothalamic tissues were homogenized in ice-cold lysis buffer containing protease and phosphatase inhibitors. Samples were lysed on ice for 30 min and then centrifuged at 12,000 rpm for 5 min at 4 °C. Supernatants were collected, and protein concentrations were determined using a BCA protein assay kit. Proteins were separated by SDS-PAGE using appropriate polyacrylamide gels based on the target protein molecular weights. Equal amounts of protein were loaded, electrophoresed, and transferred onto PVDF membranes. After blocking, membranes were incubated overnight at 4 °C with primary antibodies (ERα and MAP2K1 at 1:2000; p-ERK and p-CREB at 1:1,000; GAPDH at 1:10,000 as loading control). Subsequently, membranes were incubated with HRP-conjugated secondary antibodies for 30 min at room temperature. Protein bands were visualized using an enhanced chemiluminescence (ECL) substrate, and densitometric analysis was conducted with AlphaEaseFC software. For each sample, relative expression levels of target proteins were calculated as the ratio of their background-subtracted band intensities to GAPDH. Data represent results from three biological replicates.

### 2.15 Statistical analysis

Statistical analysis was conducted using IBM SPSS 27.0 software. Continuous variables were expressed as mean ± standard deviation (mean ± SD). For normally distributed data with homogenous variance, a one-way analysis of variance (ANOVA) was performed, followed by the least significant difference (LSD) post-hoc test for pairwise comparisons. When variances were unequal, Dunnett’s T3 test was applied instead. For non-normally distributed data, the Kruskal–Wallis H test (a non-parametric alternative) was used, with subsequent pairwise comparisons by Dunn’s test. Sample size (n) for each experiment represents the number of biologically independent animals, as specified in the figure legends. Technical replicates were averaged for each biological sample before statistical analysis. Appropriate post-hoc tests (LSD or Dunnett’s T3) were applied after ANOVA without additional correction, as these tests are specifically designed for planned pairwise comparisons within the ANOVA framework. A P-value <0.05 was considered statistically significant.

## 3 Results

### 3.1 Preparation and basic characterization of SPH

The active ingredient (SPH) was successfully prepared from authenticated seawater pearls (cultured *Pinctada martensii*) using a standardized enzymatic hydrolysis process as described. The final product was obtained as a white, lyophilized powder. To characterize its composition, protein profiles and trace element contents of SPH were analyzed. Proteomic analysis via LC-MS/MS identified peptide compositions derived from pearl matrix proteins. The total ion flow chromatogram from the mass spectrometry analysis is presented in Supplementary Figure S1. A total of 41 unique proteins were reliably identified. These proteins primarily belonged to categories including calcium-binding proteins (e.g., dermatopontin, perlucin-like proteins) and structural matrix proteins characteristic of pearl nacreous layers. A complete list of identified proteins with accession numbers, molecular weights, and sequence coverage is provided in Supplementary Table S1. Amino acid composition analysis confirmed that SPH is rich in glycine, alanine, aspartic acid/glutamic acid, and serine, consistent with the typical amino acid profile of conchiolin-like proteins. Detailed quantitative amino acid data are presented in Supplementary Table S2.

Concurrently, ICP and atomic fluorescence spectrometry analyses revealed a characteristic trace element profile of SPH (Supplementary Table S3). SPH was notably rich in calcium (Ca) and magnesium (Mg), essential macro-minerals from pearl aragonite. SPH also contained significant levels of strategically important trace elements, including zinc (Zn) and selenium (Se). Importantly, concentrations of toxic heavy metals [arsenic (As) and mercury (Hg)] were below detection limits, confirming the raw material’s safety.

### 3.2 Establishment of the PMS model and general animal health

**FIGURE 2:**
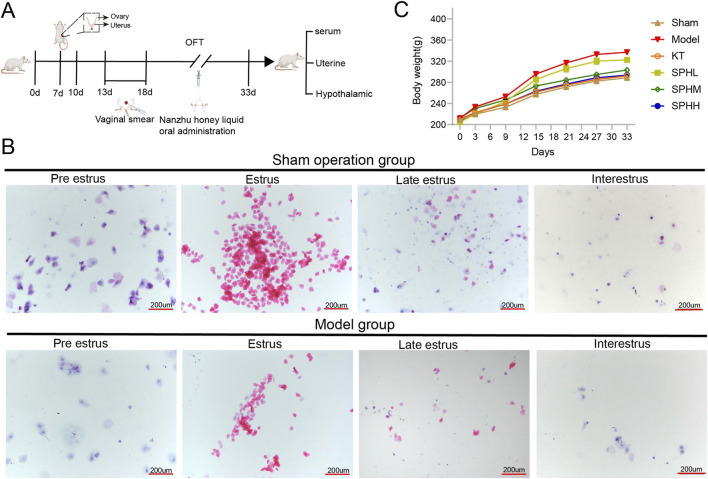
Validation of the PMS rat model. **(A)** Experimental timeline and validation of the model. **(B)** Representative vaginal smear cytology of a single rat from the Sham group across different estrous phases (H&E staining, 100×, 200 μm scale bar). **(C)** Body weight curves of rats in each group during the experimental observation period (n = 5).

A PMS model was established via surgical ovariectomy. The experimental timeline and model validation results are shown in Figure 2A. Rats in the Sham-operated group exhibited normal estrous cycles. During proestrus, nucleated epithelial cells with intact morphology and blue-stained nuclei appeared. Estrus was characterized by numerous keratinized cells with diverse morphologies arranged in clusters. Metestrus had evenly distributed keratinized cells and leukocytes, with scattered keratinized cells. Diestrus displayed abundant leukocytes accompanied by a few keratinized and epithelial cells (Figure 2B). In contrast, the Model group showed disrupted estrous cycles after ovariectomy, characterized by prolonged proestrus or estrus phases, absence of metestrus, and irregular cycle changes. Additionally, compared with the Sham-operated group, vaginal smears from the Model group showed significantly reduced cell numbers across all estrous phases (Figure 2B). Regarding general health, rats in the Model group exhibited significantly increased body weight during the postoperative observation period (Figure 2C).

### 3.3 SPH ameliorates OVX-induced behavioral deficits and normalizes organ atrophy

**FIGURE 3:**
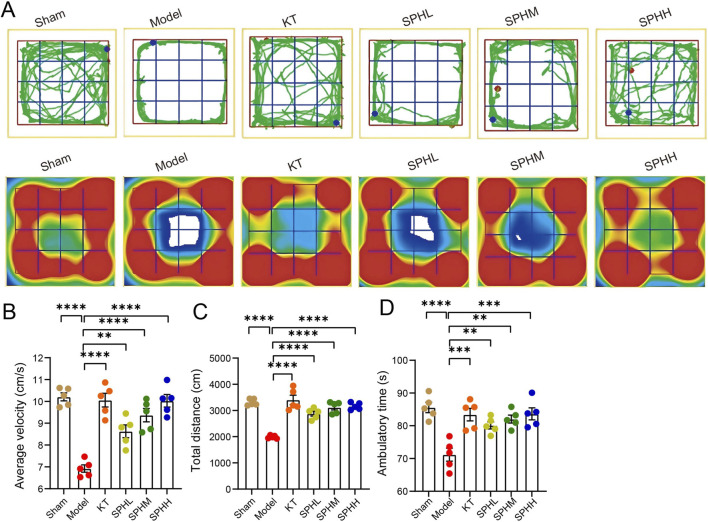
Open field locomotor trajectories. **(A)** Representative locomotor trajectories of rats from Sham, Model, KT, and SPH groups. The upper panel illustrates complete 5-min paths; the lower panel shows corresponding heatmaps of movement trajectories. **(B)** Average velocity (cm/s) **(C)** Total distance traveled (cm). **(D)** Ambulatory time (s). Compared to Sham: ^**^ *P* < 0.01, ^***^ *P* < 0.001; compared to Model, ^*^ *P* < 0.05, ^**^ *P* < 0.01, ^***^ *P* < 0.001.

PMS is often associated with reduced exploratory behavior, measurable using the open field test (Zhang et al., 2021). Compared to the Sham group, rats in the Model group showed significantly reduced average velocity, total distance traveled, and ambulatory time (all P < 0.01). These behavioral deficits were markedly reversed by treatment with the positive control (Kuntai capsules) and medium- or high-dose SPH (SPHM or SPHH), significantly increasing all three parameters (*P* < 0.05, *P* < 0.01, or *P* < 0.001; Figure 3A).

Detailed analysis confirmed that the Model group exhibited pronounced decreases in average velocity, total distance traveled, and ambulatory time relative to the Sham group (P < 0.01). Administration of the positive control or medium/high-dose SPH significantly improved these behavioral metrics (*P* < 0.05, *P* < 0.01, or *P* < 0.001; Figures 3B–D).

Uterine and hypothalamic wet weights and indices were significantly decreased in the Model group compared to the Sham group (*P* < 0.001, *P* < 0.01). Although low and medium doses of SPH (SPHL, SPHM) showed non-significant increasing trends, the SPHH and KT groups demonstrated significant recovery of these measurements (*P* < 0.001, *P* < 0.01; Table 4).

**TABLE 4:** Changes in uterine and hypothalamic wet weight and indices.

Group	Hypothalamus wet weight (mg)	Uterus wet weight (mg)	Hypothalamus inde (mg/g)	Uterus index (mg/g)
Sham	49.4 ± 4.859	404.03 ± 56.71	0.16 ± 0.013	1.33 ± 0.22
Model	36.05 ± 7.269^**^	150.15 ± 91.13^***^	0.116 ± 0.028^**^	0.51 ± 0.35^***^
KT	46.4 ± 3.489^**^	285.98 ± 75.81^***^	0.148 ± 0.012^**^	0.91 ± 0.25^***^
SPHL	39.067 ± 7.423	173.22 ± 45.72	0.124 ± 0.023	0.53 ± 0.13
SPHM	41.917 ± 7.653	177.82 ± 49.07	0.1329 ± 0.022	0.56 ± 0.16
SPHH	46.85 ± 4.556^**^	266.48 ± 84.04^***^	0.151 ± 0.014^**^	0.81 ± 0.25^***^

Compared to Sham, ^**^ *P* < 0.01, ^***^ *P* < 0.001; compared to model, ^**^ *P* < 0.01, ^***^ *P* < 0.001.

### 3.4 SPH restores hormonal homeostasis and uterine histoarchitecture

**FIGURE 4:**
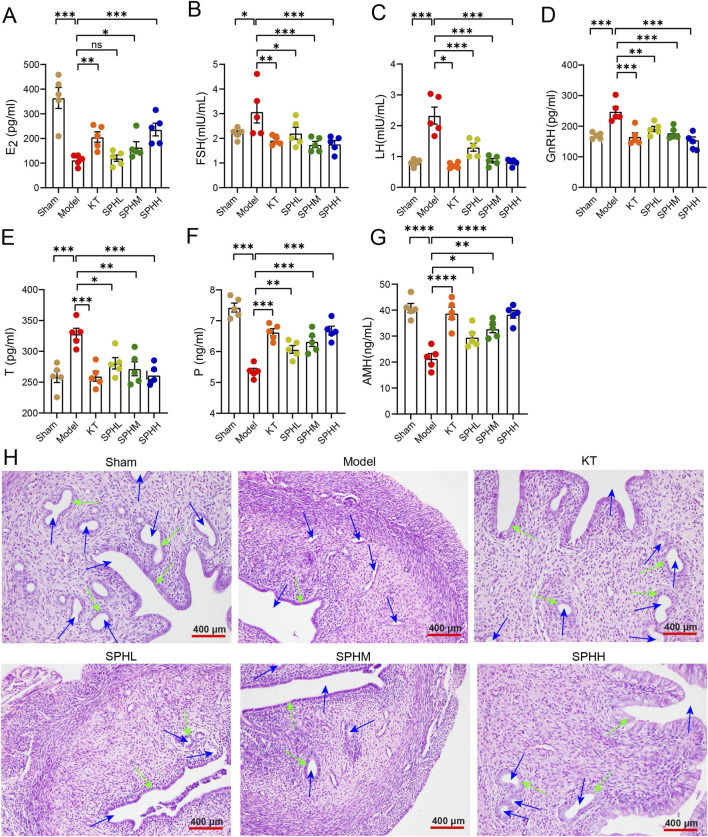
SPH restores serum sex hormone levels and uterine morphology. **(A-G)**. Serum levels of E_2_, FSH, GnRH, LH, T, P, and AMH (n = 5). Compared to the Model group, ^*^ *P* < 0.05, ^**^ *P* < 0.01, ^***^ *P* < 0.001, ^****^ *P* < 0.0001. **(H)** Uterine HE staining (n = 3; scale bar = 400 μm). Blue arrows indicate uterine cavities; orange arrows indicate simple columnar epithelial cells.

Compared with the Sham group, the Model group showed significantly reduced E_2_ and P levels (*P* < 0.001) and elevated levels of FSH, LH, and T *(P* < 0.05, *P* < 0.001). Compared to the Model group, the KT group and all SPH groups significantly increased P levels (*P* < 0.01, *P* < 0.001). Additionally, medium- and high-dose SPH groups significantly increased E_2_ levels (*P* < 0.05, *P* < 0.001). The KT and all SPH groups also significantly reduced levels of FSH, LH, GnRH, T, and AMH (*P* < 0.05, *P* < 0.01, *P* < 0.001) (Figures 4A–G).

In the Sham group, the uterine structure was well-organized, displaying distinct layers and tightly arranged cells. The uterine lumen was wide, smooth, and full, with normal endometrial morphology featuring a single layer of columnar epithelial cells. The endometrial stroma contained abundant uterine glands surrounded by numerous capillaries. In contrast, the Model group (OVX) exhibited significant pathological changes, including a narrowed uterine lumen, disorganized tissue architecture, thinned endometrium, and flattened columnar epithelial cells. The number of uterine glands was markedly reduced, glandular lumens were constricted, and capillary distribution was sparse. Treatment groups showed dose-dependent improvements in uterine morphology. Compared with the Model group, SPH-treated groups exhibited increased uterine diameter and endometrial thickness, restored columnar epithelial cell morphology, increased gland number and morphological diversity, and enhanced capillary formation. Notably, the SPHH group showed the greatest restorative effect, closely resembling the Sham group (Figure 4H).

### 3.5 Effect of SPH on oxidative stress and lipid metabolism in OVX rats

**FIGURE 5:**
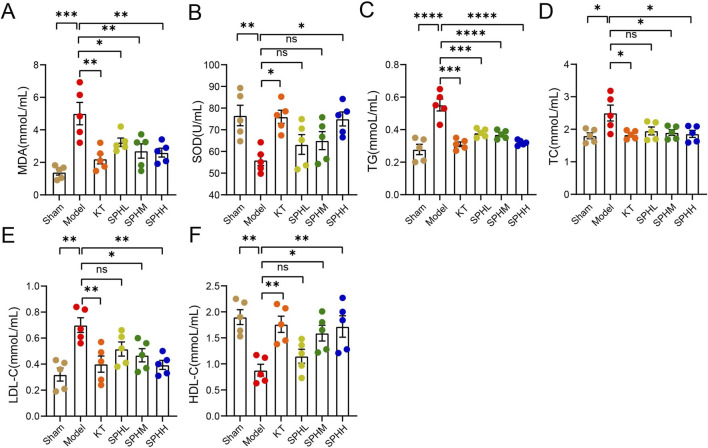
Effects of SPH on oxidative stress and lipid profiles in OVX rats. **(A,B)** Serum MDA and SOD concentrations (n = 5). **(C-F)** Serum levels of TC, TG, HDL-C, and LDL-C (n = 5). Compared with the Model group, ^*^ *P* < 0.05, ^**^ *P* < 0.01, ^***^ *P* < 0.001, ^****^ *P* < 0.0001.

PMS is associated with dyslipidemia and oxidative stress (Tan et al., 2025; Aleksejeva et al., 2025). Thus, the effects of SPH on serum MDA, SOD, and lipid levels in PMS rats were analyzed. Low, medium, and high doses of SPH significantly reduced MDA levels (*P* < 0.05, *P* < 0.01), while the high dose significantly increased SOD activity (*P* < 0.05, Figures 5A,B). Compared with the Sham group, rats in the Model group showed elevated TC, TG, and LDL-C levels and decreased HDL-C levels (Figures 5C–F). Medium and high doses of SPH significantly reduced TC, TG, and LDL-C levels (*P* < 0.05, *P* < 0.01, *P* < 0.0001, Figures 5C–E) and significantly increased HDL-C levels (*P* < 0.05, *P* < 0.01, Figure 5F). These findings indicate SPH alleviates PMS by regulating oxidative stress and lipid metabolism, with medium and high doses showing greater efficacy in restoring lipid balance.

### 3.6 SPH upregulates mRNA expression of ERα/MAPK/CREB pathway components

**FIGURE 6:**
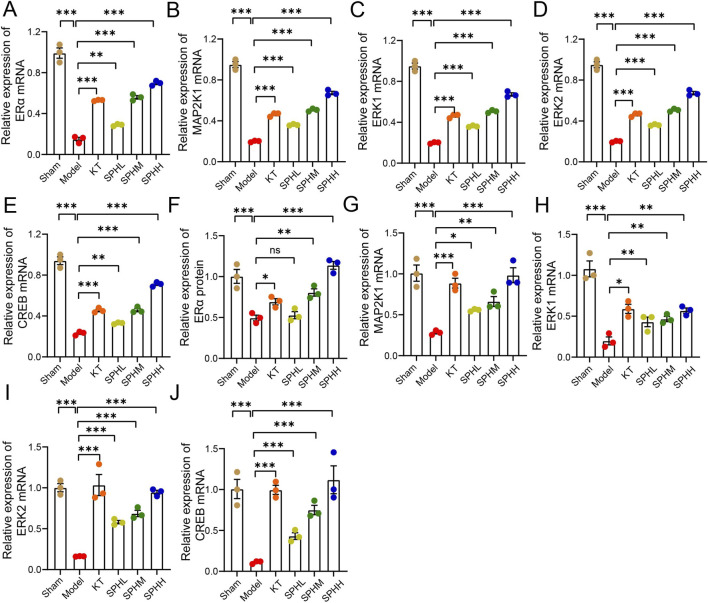
SPH upregulates mRNA expression of ERα/MAPK/CREB pathway components in OVX rats. **(A–E)** Relative mRNA levels in uterine tissues for **(A)** ERα, **(B)** MAP2K1, **(C)** ERK1, **(D)** ERK2, and **(E)** CREB. **(F–J)** Relative mRNA levels in hypothalamic tissue for **(F)** ERα, **(G)** MAP2K1, **(H)** ERK1, **(I)** ERK2, and **(J)** CREB. Data are normalized to β-actin. Compared with the Model group, ^*^ *P* < 0.05, ^**^ *P* < 0.01, ^***^ *P* < 0.001, ^****^ *P* < 0.0001.

Compared to the Sham group, mRNA expression levels of ERα, MAP2K1, ERK1/2, and CREB in uterine tissues were significantly decreased in the Model group (*P* < 0.001). Treatment with KT or low-, medium-, and high-dose SPH (SPHL, SPHM, SPHH) significantly increased these transcripts compared with the Model group (*P* < 0.01, *P* < 0.001) (Figures 6A–E).

Similarly, compared to the Sham group, hypothalamic tissues in the Model group displayed significantly reduced mRNA expression of ERα, MAP2K1, ERK1/2, and CREB (*P* < 0.001). The KT group and medium- and high-dose SPH groups showed significant upregulation of these genes (*P* < 0.01, *P* < 0.001). Additionally, the low-dose SPH group significantly increased hypothalamic mRNA expression of MAP2K1, ERK1/2, and CREB (*P* < 0.05, *P* < 0.01, *P* < 0.001) (Figures 6F–J).

### 3.7 SPH alters protein expression and phosphorylation of the ERα/MAPK/CREB pathway

**FIGURE 7:**
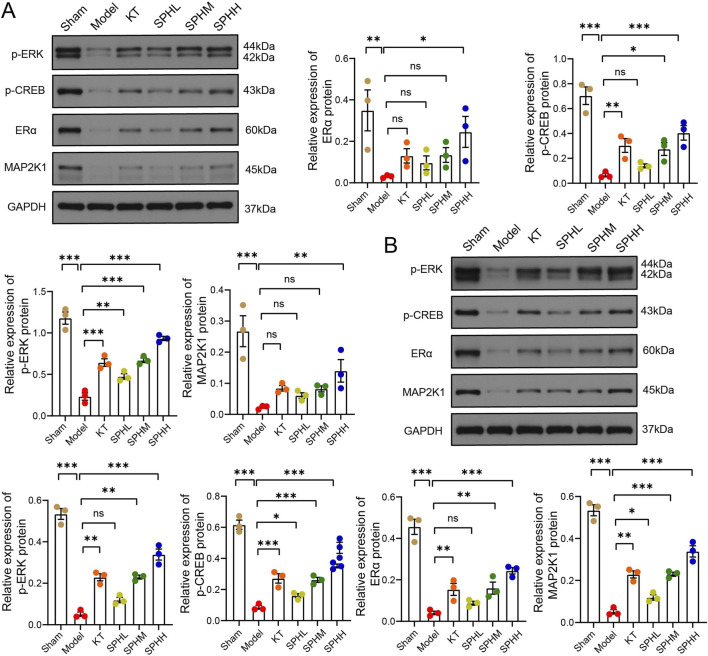
SPH alters protein expression and phosphorylation of the ERα/MAPK/CREB pathway in OVX rats. **(A)** Uterine protein levels of ERα, p-CREB, p-ERK, and MAP2K1. **(B)** Hypothalamic protein levels of p-ERK, p-CREB, ERα, and MAP2K1. GAPDH were used as a loading control. Data are presented as mean ± SD. Compared with the Model group, ^*^ *P* < 0.05, ^**^ *P* < 0.01, ^***^ *P* < 0.001, ^****^ *P* < 0.0001.

Western blot analysis demonstrated significantly reduced protein expression levels of ERα, MAP2K1, p-ERK, and p-CREB in uterine tissues of the Model group compared to the Sham-operated group (*P* < 0.01; Figure 7A). SPH treatment reversed these changes in a dose-dependent manner. The high-dose group significantly increased expression of all four proteins (ERα, MAP2K1, p-ERK, and p-CREB) relative to the Model group (*P* < 0.05, *P* < 0.01). The medium-dose group significantly elevated p-ERK and p-CREB expression (*P* < 0.01), while the low-dose group specifically enhanced MAP2K1 and p-CREB levels (*P* < 0.05). Similarly, the positive control (Kuntai capsules) significantly increased expression of p-ERK and p-CREB (*P* < 0.01).

Compared with the Sham-operated group, protein expression of ERα, MAP2K1, p-ERK, and p-CREB in hypothalamic tissues of the Model group rats was significantly decreased (*P* < 0.001). In contrast, compared with the Model group, the KT group and medium- and high-dose SPH groups significantly elevated hypothalamic protein expression of ERα, MAP2K1, p-ERK, and p-CREB (*P* < 0.01, *P* < 0.001). Additionally, the low-dose SPH group significantly increased hypothalamic MAP2K1 and p-CREB protein expression (*P* < 0.05) (Figure 7B).

## 4 Discussion

PMS results from hormonal imbalance due to ovarian dysfunction during reproductive aging. Its main pathophysiological features include significantly decreased secretion of E_2_ and P, along with compensatory elevations in gonadotropins (FSH, LH, and GnRH). These endocrine disruptions cause endometrial atrophy and impair the hypothalamic-pituitary-ovarian (HPO) axis (Devillers et al., 2022; Kling et al., 2019). Using an OVX rat model of PMS, this study systematically explored how SPH, a hydrolysate derived from sustainably sourced, cultured seawater pearls, mitigates histopathological damage in uterine and hypothalamic tissues by modulating the MAPK/CREB signaling pathway. Findings indicate that SPH significantly improves PMS-like symptoms, including behavioral deficits, organ atrophy, hormonal imbalance, oxidative stress, and dyslipidemia. These improvements correlate with restored ERα/MAPK/CREB signaling in the uterus and hypothalamus.

In the OVX model, serum E_2_ and P levels decreased, while FSH, GnRH, LH, T, and AMH increased (Davis et al., 2023; Stanczyk et al., 2019). SPH treatment effectively reversed these changes, confirming its broad endocrine-regulating potential (Xiong et al., 2022; Tian et al., 2019). Histopathological evaluation of the uterus in the Model group showed glandular reduction, epithelial atrophy, and increased leukocyte infiltration. SPH treatment reversed these abnormalities and improved estrous cycles. Behavioral assessments in the open-field test indicated enhanced activity. However, future studies should use anxiety-specific models (e.g., elevated plus maze) to confirm efficacy against perimenopause-related anxiety. Mechanistically, the hypothalamus is a critical brain region regulating emotional and stress responses. This study confirmed that SPH treatment is associated with increased expression of ERα and altered activity of downstream MAPK/CREB signaling. Phosphorylation of CREB (p-CREB) is a pivotal event regulating the expression of neuroplasticity-related genes. Findings suggest that SPH may alleviate perimenopause-related symptoms by modulating the hypothalamic ERα/MAPK/p-CREB pathway, potentially influencing neurotrophic factor expression. This hypothesis provides an integrated view linking peripheral and central effects. ERα is crucial for mediating E_2_ signals (Ye et al., 2022; Ohlsson et al., 2020). Following OVX, rapid declines in E_2_ reduce ERα sensitivity (Wu et al., 2011; Eriksson, 1982). Our results show SPH elevates ERα protein levels alongside increased serum E_2_ levels. Thus, PMS symptom alleviation by SPH may involve restoration of E_2_ signaling via ERα, consistent with findings from other herbal extracts (Stute et al., 2022; Zhou et al., 2019; Lee et al., 2021).

Beyond direct modulation of the ERα/MAPK/CREB axis, PMS treatment increasingly recognizes immune-endocrine crosstalk and inflammatory signaling. Recent studies highlight uterine immune cell dysregulation (Kang et al., 2024) and systemic inflammation in menopausal pathology. Several phytochemicals modulate inflammatory pathways via nuclear receptors (Wu et al., 2025), and extracellular vesicle signaling has emerged as key in suppressing inflammation (Yu et al., 2025). Although this study primarily addressed hormonal and kinase pathways, improved uterine morphology and potential reductions in oxidative stress (a pro-inflammatory trigger) suggest SPH may possess immunomodulatory effects. Future studies measuring inflammatory cytokines (e.g., TNF-α, IL-6) and exploring connections with translational biomarkers of reproductive immune-endocrine imbalance (Klein and Flanagan, 2016; Chrousos, 1995) could clarify these mechanisms.

Notably, SPH significantly reduced oxidative stress and dyslipidemia, contributing to its therapeutic profile. MAP2K1 and ERK are critical components of the MAPK pathway, regulating cell proliferation and differentiation (Miningou and Blackwell, 2020). CREB is a key transcription factor downstream of ERK (Chowdhury et al., 2023). Treatment groups showed increased expression of p-ERK and p-CREB in hypothalamic and uterine tissues compared to the Model group. This aligns with reports that other formulations activate this pathway in PMS models (Guo et al., 2022; Li et al., 2019). Thus, modulation of MAPK/CREB signaling may represent a common therapeutic mechanism among multiple PMS interventions.

In summary, this study demonstrates SPH—a hydrolysate of pearls from sustainably cultured *Pinctada martensii*—effectively alleviates PMS symptoms in OVX rats. The therapeutic effects are associated with restored hormonal balance, reduced oxidative stress, improved lipid metabolism, and modulation of the ERα/MAPK/CREB signaling pathway in both uterine and hypothalamic tissues, suggesting an integrated neuroendocrine regulatory mechanism. The novel formulation enhances its ethnopharmacological relevance. However, key limitations should be acknowledged: mechanistic interpretations rely on correlational protein expression data without functional inhibition experiments (e.g., pathway inhibitors), and phosphorylation was assessed at a single endpoint, reflecting sustained rather than acute changes. Future research should employ loss-of-function and kinetic analyses to confirm causality. Additionally, integrating these results with existing literature on similar traditional preparations would further enhance the discussion. Advanced omics-based approaches are warranted to comprehensively elucidate the mechanisms underlying this promising formulation.

## Data Availability

The raw data supporting the conclusions of this article will be made available by the authors, without undue reservation.
