# Supplementary material for: Seawater pearl hydrolysate alleviates perimenopausal syndrome by modulating hypothalamic and uterine ERα/MAPK/CREB signaling in ovariectomized rats
Source: Front Pharmacol. 2026 Feb 5;17:1749728. doi: 10.3389/fphar.2026.1749728 (PMC12916607; doi:10.3389/fphar.2026.1749728)
Supplement: Supplementary file 1 [file Supplementaryfile1.docx]

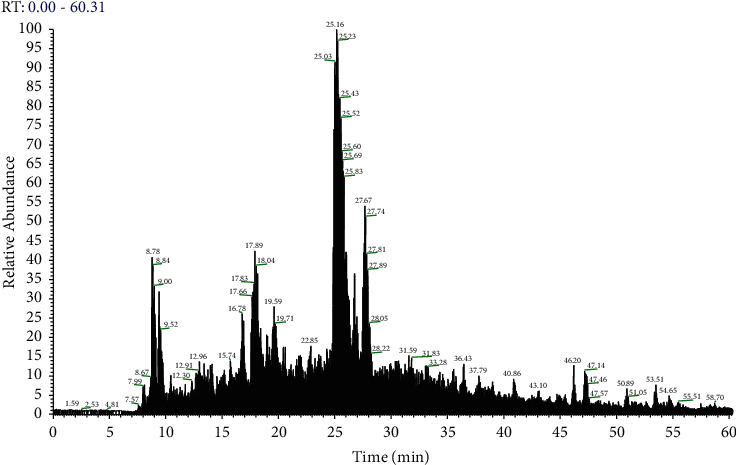


**Fig S1 Total ion flow chromatogram of protein mass spectrometry of SPH**

**Table S1. The proteins are detected in the SPH proteomic analysis**

| **Protein IDs** | **Majority protein IDs** | **Protein name** | **gene** | **Score** | **Intensity** |
| --- | --- | --- | --- | --- | --- |
| G9MD31;REV__G9MD31 | G9MD31 | MSI60-related protein | MSI60RP | 203.54 | 45096000 |
| M3VMM6;M3VMN1;I7GPT9 | M3VMM6;M3VMN1 | Insoluble matrix protein | MSI80 | 147.11 | 42832000 |
| O02402;M3VMM8 | O02402;M3VMM8 | Insoluble protein | N/A | 136.46 | 465860000 |
| A1IHF0 | A1IHF0 | Tyrosinase-like protein 1 | Pfty1 | 110.45 | 1596600 |
| L8B691;L8B677;L8B659;L8B644;L8B639;L8B620;L8B611;L8B5Y5;L8B5W5;L8B5W0;L8B5V5;L8B5V2;L8B5T6;L8B2R0;L8B673;L8B5W9 | L8B691;L8B677;L8B659;L8B644;L8B639;L8B620;L8B611;L8B5Y5;L8B5W5;L8B5W0;L8B5V5;L8B5V2;L8B5T6;L8B2R0;L8B673 | Nacre protein | N19 | 94.365 | 106890000 |
| A0A0K1RN13 | A0A0K1RN13 | N-U5 | N/A | 68.801 | 21334000 |
| L8B6K4;L8B689;L8B725;L8B6N2;L8B6H1;L8B6B5;L8B6B2;L8B3R6 | L8B6K4;L8B689;L8B725;L8B6N2;L8B6H1;L8B6B5;L8B6B2;L8B3R6 | Nacre protein | N16 | 36.601 | 25549000 |
| I7H5B0;X2GHQ9;L8B6Y0;L8B6T0;L8B6L4;L8B6K6;L8B6E5;L8B6A3;L8B621;L8B3M5;L8B3J4;L8B3G5;L8B3D9;I7H832;I7H822;I7H800;I7H7U3;I7H5G5;I7H5F7;I7H5E4;I7H598;I7H1K7;I7H1J2;I7GVW1;I7GVW0;I7GQ94;I7GQ68 | I7H5B0;X2GHQ9;L8B6Y0;L8B6T0;L8B6L4;L8B6K6;L8B6E5;L8B6A3;L8B621;L8B3M5;L8B3J4;L8B3G5;L8B3D9;I7H832;I7H822;I7H800;I7H7U3;I7H5G5;I7H5F7;I7H5E4;I7H598;I7H1K7;I7H1J2;I7GVW1;I7GVW0;I7GQ94;I7GQ68 | Nacre protein | N16 | 30.121 | 8936600 |
| A0ZSF2;Q27908;D1MYW1;A0A286K025;A0A286K024 | A0ZSF2;Q27908;D1MYW1;A0A286K025;A0A286K024 | Nacrein-like protein F | N/A | 20.945 | 3677000 |
| L8B608;S6BUC1;L8B6C6;L8B6B1;L8B699;L8B695;L8B669;L8B663;L8B648;L8B646;L8B625;L8B602;L8B5Z7;L8B5Z5;L8B5Y7;L8B5Y4;L8B5X0;L8B5U3;L8B5T4;L8B5S3;L8B5Q1;L8B300;L8B2T7;L8B2Q6;G1K3U3;G1K3U2;G1K3U0;G1K3T9;G1K3T8;G1K3T7;G1K3T6;G1K3T5;G1K3T4;G1K3T2;G1K3T1;G1K3T0;G1K3S8;G1K3S7;A7VMH6;L8B2R4 | L8B608;S6BUC1;L8B6C6;L8B6B1;L8B699;L8B695;L8B669;L8B663;L8B648;L8B646;L8B625;L8B602;L8B5Z7;L8B5Z5;L8B5Y7;L8B5Y4;L8B5X0;L8B5U3;L8B5T4;L8B5S3;L8B5Q1;L8B300;L8B2T7;L8B2Q6;G1K3U3;G1K3U2;G1K3U0;G1K3T9;G1K3T8;G1K3T7;G1K3T6;G1K3T5;G1K3T4;G1K3T2;G1K3T1;G1K3T0;G1K3S8;G1K3S7;A7VMH6 | Nacre protein | N19 | 15.849 | 24875000 |
| A0A0P0LE82;A0A0K1RMW9 | A0A0P0LE82;A0A0K1RMW9 | Alveoline-like protein | N/A | 12.395 | 2953000 |
| L8AX46 | L8AX46 | Vasa-like gene-4 | vlg4 | 10.259 | 0 |
| A0A194APP9 | A0A194APP9 | Uncharacterized protein | N/A | 9.7735 | 69137000 |
| A0A194AMA0 | A0A194AMA0 | Putative aldehyde dehydrogenase；mitochondrial-like protein | N/A | 8.7645 | 0 |
| A0A1P8P0H8 | A0A1P8P0H8 | Hexosyltransferase | N/A | 8.3377 | 164320 |
| A0A194APW9 | A0A194APW9 | Putative serine and glycine-rich matrix protein 1 | N/A | 8.0478 | 0 |
| A0A194ALQ1 | A0A194ALQ1 | Elongation factor 1-alpha | N/A | 7.7658 | 1175200 |
| R4IS90 | R4IS90 | Tyrosine-protein kinase receptor | irr | 7.6773 | 550500 |
| L8B2P8;G1K3U4 | L8B2P8;G1K3U4 | Nacre protein | N19 | 7.2956 | 1666900 |
| C7G0B5 | C7G0B5 | Protein PIF | N/A | 7.0349 | 5246700 |
| A0A0K1RN15 | A0A0K1RN15 | N-U10 | N/A | 7.0069 | 4156600 |
| A0A0P0M060 | A0A0P0M060 | TSP1-containing protein | N/A | 6.9588 | 0 |
| A0A0K1RMU0 | A0A0K1RMU0 | N-U2 | N/A | 6.8097 | 565130 |
| A0A194AJH2 | A0A194AJH2 | Uncharacterized protein | N/A | 6.4898 | 1313800 |
| A0A0K1RNE9 | A0A0K1RNE9 | Amine oxidase |  | 6.3251 | 2407800 |
| A0A194ANT4 | A0A194ANT4 | Putative regucalcin-like protein | N/A | 6.2493 | 1862800 |
| B6CI69;A0A068CMR3 | B6CI69;A0A068CMR3 | Shematrin 2 | N/A | 6.1737 | 3102800 |
| A0A194ALS0 | A0A194ALS0 | Uncharacterized protein |  | 6.1508 | 648210 |
| A0A194AM17 | A0A194AM17 | Putative 60S ribosomal protein L7-like protein | N/A | 6.1356 | 0 |
| Q17TZ1 | Q17TZ1 | Alkaline phosphatase | N/A | 6.1347 | 3686300 |
| A0A194AQK3 | A0A194AQK3 | Amine oxidase | N/A | 6.104 | 6722800 |
| A0A194AMQ9 | A0A194AMQ9 | Uncharacterized protein | N/A | 6.0738 | 819020 |
| C9WMI0 | C9WMI0 | Ribosomal protein L37a | N/A | 6.0702 | 23422 |
| A0A0N7J6H7 | A0A0N7J6H7 | Copper/zinc superoxide dismutase | N/A | 6.0508 | 0 |
| A0A191Z434 | A0A191Z434 | Bone morphogenetic protein 3 | *N/A* | 6.0093 | 1668500 |
| A0A194AN95 | A0A194AN95 | 40S ribosomal protein S30 | *N/A* | 6.0081 | 0 |
| A0A194AL43 | A0A194AL43 | Uncharacterized protein | *N/A* | 6.005 | 8256900 |
| A0A194AN67 | A0A194AN67 | Uncharacterized protein | *N/A* | 5.984 | 0 |
| A0A194AJT7 | A0A194AJT7 | Putative methenyltetrahydrofolate synthase domain-containing protein-like protein | *N/A* | 5.9774 | 0 |
| A1IHF1 | A1IHF1 | Tyrosinase-like protein 2 | *Pfty2* | -2 | 1381500 |
| L8AZT9 | L8AZT9 | Vasa-like gene-1 | *vlg1* | -2 | 0 |

**Table S2 Amino acid composition of the SPH**

| Amino Acid | Concentration (µg/ml)) |
| --- | --- |
| **Alanine** | 0.223 |
| **Arginine** | 0.436 |
| **Glycine** | 0.188 |
| **Histidine** | 0.524 |
| **Isoleucine** | 0.328 |
| **Lysine** | 0.457 |
| **Methionine** | 0.373 |
| **Phenylalanine** | 0.413 |
| **Proline** | 0.288 |
| **Serine** | 0.263 |
| **Threonine** | 0.298 |
| **Tyrosine** | 0.453 |
| **Valine** | 0.293 |

**Table S3 Contents of trace elements in SPH (mg/kg).**

| S | P | Fe | Mn | Cu | Na | Mg | Zn | Sr | Se | As | Hg |
| --- | --- | --- | --- | --- | --- | --- | --- | --- | --- | --- | --- |
| 7.46 | 74.3 | 26.3 | 9.66 | 2.03 | 5943 | 631 | 47.6 | 1003 | 0.046 | 3.50 | 0.0 |
